# Mechanisms of dyspnoea and exercise intolerance in smokers with preserved ratio impaired spirometry

**DOI:** 10.1007/s00421-026-06244-3

**Published:** 2026-06-06

**Authors:** Alice Scussel, Nathalia Galvagni Rodrigues, Artur Zanelatto Santos, Laura Corrêa de Barros Trombin, Amanda Calage Pinto, Litiele Evelin Wagner, Marli Maria Knorst, James Dean, Dave Singh, Danilo Cortozi Berton

**Affiliations:** 1 Programa de Pós-Graduação em Ciências Pneumológicas, Universidade Federal do Rio Grande do Sul (UFRGS), Unidade de Fisiologia Pulmonar, Hospital de Clínicas de Porto Alegre (HCPA), Rua Ramiro Barcelos, 2350, Prédio A, Sala 2050, Porto Alegre, RS Brazil; 2 Medicines Evaluation Unit, an IQVIA Business, Manchester, UK; 3 Division of Immunology, Immunity to Infection and Respiratory Medicine, School of Biological Sciences, Faculty of Biology, Medicine and Health, The University of Manchester, Manchester University NHS Foundation Trust, Manchester, UK

**Keywords:** COPD, Preserved Ratio Impaired Spirometry (PRISm), Smokers, Spirometry, Exercise test, Dyspnoea

## Abstract

**Purpose:**

Smokers with preserved ratio impaired spirometry (PRISm) present reduced forced expiratory volume in the first second (FEV₁ < 80% predicted) but preserved FEV_1_/forced vital capacity (FVC) ≥ 0.7.  Although they do not meet diagnostic criteria for chronic obstructive pulmonary disease (COPD), they often report respiratory symptoms and exercise intolerance. We aimed to compare sensory and physiological responses to exercise between smokers with PRISm and matched healthy controls. Patients with mild-to-moderate COPD were included as a reference group.

**Methods:**

In this cross-sectional study, smokers (> 10 pack-years) from a specialized outpatient clinic were evaluated. Thirteen participants (11 females; 62.4 ± 7.7 years) with PRISm and 13 age- and sex-matched patients with mild-to-moderate COPD (FEV₁/FVC < 0.7; FEV₁ > 50% predicted) underwent incremental cardiopulmonary exercise testing on a cycle ergometer. Data from healthy nonsmoking controls were obtained from previous studies.

**Results:**

PRISm and COPD groups had similarly reduced FEV₁ (~ 72% predicted) compared to controls (97% predicted), whereas FVC was lower in PRISm (73% predicted) than in COPD and controls (~ 95% predicted). Both patient groups showed higher ventilation, reduced ventilatory reserve, and greater dyspnoea at a given submaximal workload versus controls. Increased tidal volume/inspiratory capacity ratios were also observed, contributing to greater dyspnoea for a given ventilation and lower peak work rate (42 ± 16 vs. 68 ± 11 vs. 94 ± 17% predicted, respectively; all *P* < 0.05).

**Conclusion:**

Smokers with PRISm exhibit increased dyspnoea and reduced exercise tolerance compared to healthy controls, largely driven by excessive ventilatory demand and reduced ventilatory capacity during submaximal exercise.

## Introduction

The concept of preserved ratio impaired spirometry (PRISm) was first introduced by Wan and colleagues in 2014 to classify people with an FEV₁ less than 80% of predicted or less than lower limit of normal (LLN) in the presence of a normal FEV₁/forced vital capacity (FVC) ratio (i.e., FEV₁/FVC ≥0.7 or more than LLN) in an observational study of current or former smokers aged 45-80 years with ≥10 pack-years of smoking (Wan et al. 2014). Since then, PRISm is a topic of increased investigation due to its significant prevalence (⁓12%) (Robertson et al. 2025) and association with respiratory symptoms, cardiovascular disease, metabolic syndrome and increased mortality (Wan et al. 2021, 2014, 2018; Wijnant et al. 2020). As corollary, the PRISm concept was first introduced in the Global Initiative for Chronic Obstructive Lung Disease (GOLD) report in 2023 as a pre-COPD condition at risk of developing airflow obstruction over time, representing an opportunity for COPD prevention, early diagnosis and prompt therapeutic intervention (Agustí et al. 2023a).

A distinctive feature of PRISm that has gained attention is the greater exertional dyspnoea and lower exercise capacity (Phillips et al. 2024), physical performance (Anami et al. 2021), more frequent respiratory symptoms and poorer quality of life (Zhou et al. 2025). It was shown that exercise performance was mainly influenced by the magnitude of prevailing inspiratory mechanical constraints, regardless of sex, body mass index, FEV_1_ and resting gas exchange capacity (diffusing capacity of the lungs for carbon monoxide; DL_CO_). It means that, because of a lower FVC and inspiratory capacity (IC) at rest, constraints for further tidal volume (VT) expansion was met at lower workload and ventilation compared to healthy controls (Phillips et al. 2024). With progressively higher VT/IC ratios at low workloads, the VT becomes positioned at the upper less compliant portion of the respiratory system’s pressure–volume relationship. Even at relatively light-to-moderate exercise, elastic loading of the inspiratory muscles and inspiratory neural drive may increase to high levels to sustain ventilation commensurate with metabolic requirements (Faisal et al. 2016; James et al. 2022). The excessive inspiratory neural drive is associated with higher dyspnoea at relatively low ventilation (Faisal et al. 2016; James et al. 2022; Jolley et al. 2015; Schaeffer et al. 2014), even in symptomatic smokers without (Elbehairy et al. 2016) or just with mild COPD (Guenette et al. 2014).

However, a significant heterogeneity in the definitions and nomenclature used for impaired lung function characterized by proportionate reductions in FEV_1_ and FVC exists. It has also been described as restrictive (Backman et al. 2016; Guerra et al. 2010; Mannino et al. 2012), non-specific (Hyatt et al. 2009; Iyer et al. 2011), or unclassified spirometry (Wan et al. 2011). Accordingly, a variety of different conditions, including some with ominous prognosis, have been reported to be associated with this pattern (Godfrey & Jankowich 2016; Stanojevic et al. 2022). Not surprisingly, there are a plenty number of population-based epidemiological studies (Cadham et al. 2024; Higbee et al. 2022; Wallström et al. 2025; Wan et al. 2021) linking the PRISm pattern with increased rates of hospitalization and mortality. They should be viewed with caution, however, since concomitant comorbidities may have caused the PRISm pattern, or that this pattern, in fact, represented an actual restrictive ventilatory defect (i.e., total lung capacity (TLC) < lower limit of normal (LLN)) (Knox-Brown et al. 2022). Accordingly, among the 59 patients in the main study exploring the mechanisms underpinning dyspnoea and exercise intolerance in patients with PRISm (Phillips et al. 2024), nine (15%) presented with restriction. Furthermore, the 20 never-smokers (representing 1/3 of the sample) showed similar lung structure, resting pulmonary function and exercise characteristics to the 39 ever-smokers (including 16 active smokers), suggesting no effect of smoking history on lung function within the PRISm group and raising the concern about an undetected significant pathophysiological comorbidity in these subjects.

We reasoned that smokers with PRISm, and without confounding comorbidities causing this ventilatory impairment, mainly present such impairment due to insidious peripheral airway dysfunction. Such abnormality would increase neural drive to the inspiratory muscles (Guenette et al. 2014), while inducing some degree of ventilatory constraint due to reduced estimated maximal voluntary ventilation (eMMV = FEV_1_ x 40) (Berton et al. 2023) and IC at rest (Faisal et al. 2016). To test this hypothesis, we compared perceptions of dyspnoea and detailed ventilatory responses during symptom-limited incremental exercise between a selected group of smokers with PRISm and age- and sex-matched healthy never-smokers. Patients with mild-to-moderate COPD were also included as a reference group for abnormal ventilatory and sensory responses to exercise.

## Methods

### Study design

Cross-sectional, single-center study with data collected prospectively between January/2023 and July/2025. Patients at our smoking cessation and COPD outpatient clinic who underwent spirometry were evaluated for inclusion. Clinical characteristics (including modified Medical Research Council dyspnoea score (mMRC), COPD assessment test (CAT), chest image, and, when available, echocardiography) and resting pulmonary function tests (spirometry, plethysmography, and DL_CO_) were reviewed from electronic medical records. Pulmonary function tests were performed with a computerized system (MasterScreen Body/Diffusion System®, Jaeger, Hoechberg, Germany) according to 2005 American Thoracic Society and European Respiratory Society standards and presented as absolute and percentage of predicted values (Global Lung Function Initiative (GLI) reference values). Resting pulmonary function tests within 12 months of the planned study visit were used.

Once patients fulfilled the inclusion and exclusion criteria, they were invited to a single study visit when they performed an incremental cardiopulmonary exercise test (CPET) with detailed measurements of ventilatory and sensory-perceptual responses. This study received ethical approval from our institutional Ethical Committee (N◦ 2022–0562/CAAE 65934122.3.0000.5327) and all participants signed written informed consent.

### Participants

All smokers over the age of 18 years with at least 3 months of clinical stability, with a history of smoking (current or former) > 10 pack-years, were considered for inclusion. Individuals who had cognitive impairment, orthopedic or neurodegenerative disease, advanced or recently diagnosed neoplastic disease (except skin cancer), heart failure, morbid obesity (body mass index (BMI) > 40 kg/m^2^), TLC ≤ LLN and/or other conditions (pulmonary hypertension, asthma, interstitial lung disease, kidney transplant) that the researchers considered to have a potential impact on exercise performance were excluded.

PRISm was defined by post-bronchodilator FEV_1_/FVC ≥ 0.7 and FEV_1_ < 80% of predicted. Smokers with post-bronchodilator FEV_1_/FVC < 0.7 were classified as having COPD and considered for inclusion in the presence of mild (GOLD 1 = FEV_1_ ≥ 80% of predicted) or moderate (GOLD 2 = 50% ≤ FEV_1_ < 80% of predicted) spirometry severity (Agustí et al. 2023a). The criteria for COPD and PRISm was confirmed by spirometry performed immediately prior to CPET.

The healthy nonsmoking controls were retrieved from a database including subjects who had already participated in previous studies from our center that performed similar CPET evaluation (Neder et al. 2020).

The COPD and healthy non-smoking subjects were matched to the PRISm group according to age, sex, and BMI.

### Procedures

#### Cardiopulmonary Exercise Test (CPET)

The exercise tests were carried out on an electronic brake cycle ergometer (Corival, Lode®, Groningen, the Netherlands) using a Vmax Encore® computerized system (Carefusion, Yorba Linda, CA, USA). Patients were requested to sustain the use of their regular medication and avoid using short-acting bronchodilators 6 h before the test. The exercise protocol consisted of a steady-state rest period of 5 min, a subsequent 2-min unloaded pedaling, followed by an incremental ramp exercise phase of 5-10Wmin (patients) or 10-15W/min (controls) until exhaustion according to international standardization (Radtke et al. 2019). Pedaling frequency was maintained around 60 revolutions per minute. Standard measurements of oxygen uptake (V̇O_2_), carbon dioxide production (V̇CO_2_), minute-ventilation (V̇E), breathing frequency (*f*) and VT were collected breath‐by‐breath. Peak V̇E and V̇O₂ were calculated using the last 20-s bin value at exercise termination. ECG was continuously monitored (Cardiosoft®, GE HealthCare, Milwaukee, USA) as well as oxyhemoglobin saturation (SpO_2_) (Takaoka Oxicap®, Sao Paulo, Brazil). Shortness of breath and leg effort were questioned every 2 min using the 10‐point Borg scale immediately before the IC measurements. All participants received consistent instructions to perform a maximal inspiration from a stable end expiratory lung volume (EELV; at least four breaths) to TLC. Verbal encouragement was provided during full inspiration. For every IC maneuver, the breathing pattern and the absence of significant EELV drift were assessed. A repeatability criterion of 5% or 100 mL (whichever was greater) was used, and the larger value was recorded at each time point. At least three maneuvers at rest and two maneuvers during exercise were performed. Additional maneuvers were conducted whenever the acceptability or repeatability criteria were not met (Guenette et al. 2013). Standard cardiorespiratory parameters were exported as 20-s averages. A maximal exercise test was defined when at least one of the following criteria was achieved: (1) respiratory exchange ratio > 1.05; and/or (2) heart rate > 85% of the predicted value; and/or (3) ⩒E/ estimated maximal voluntary ventilation (eMVV = FEV_1_ × 40) > 0.85 and/or d) VT/IC > 0.70 (Berton et al. 2021; Radtke et al. 2019).

### Statistical analysis

Based on a previous study in mild COPD (Elbehairy et al. 2015), we estimated that a sample size of at least 11 subjects in each group would be large enough to detect significant between-group differences in ventilatory and sensory responses to incremental exercise.

Continuous data are presented as mean ± standard deviation (SD) or median (25^th^–75^th^ percentile) according to data distribution, and categorical data as number (%). The distribution of the data was evaluated using the Shapiro–Wilk test. Baseline and peak exercise variables of the three groups were compared by χ^2^ or Fisher’s exact tests, analysis of variance (ANOVA) or Kruskal–Wallis test (when the data was not normally distributed). A Bonferroni post-hoc test was used to perform pairwise comparisons to identify which specific groups have significant differences when the ANOVA or Kruskal–Wallis tests were statistically significant. Exercise testing parameters were analyzed within and between groups at rest and standardized work rates and V̇E using a generalized estimating equation model with post hoc Bonferroni adjustment for multiple comparisons. The SPSS package (v. 18) was used for the statistical analyses. A *p* < 0.05 indicated statistical significance for all tests.

## Results

**Fig. 1:**
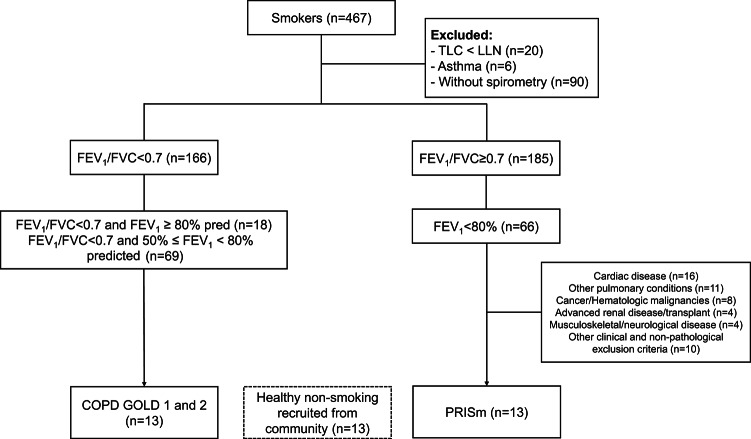
Flowchart of participants’ inclusion. *TLC* total lung capacity, *LLN*, lower limit of normal, *FEV*_*1*_, forced expired volume in the first second, *FVC* forced vital capacity; *GOLD* Global Initiative for Obstructive Lung Disease, *PRISm* preserved ratio impaired spirometry

Four hundred and sixty-seven patients referred to our smoking cessation clinic were pre-screened. After considering the inclusion and strict exclusion criteria, 13 patients accepted to perform an incremental CPET and were included in the PRISm group. As per protocol, all these subjects presented with TLC > LLN, indicating the absence of a restrictive ventilatory defect. From this pre-screened population, 13 patients with mild to moderate COPD were age and sex-matched, and selected to compose the COPD group (Fig. 1). The control group consisted of 13 healthy never-smoking individuals.

There was no significant difference in sex, age and BMI among groups. The PRISm group had a higher rate of active smokers and tended to have more smoking pack-years than the COPD group. Both groups of patients presented with a similar high burden of dyspnoea (mMRC scale) and overall respiratory symptoms (CAT score) (Table 1). Non-statistically significant higher rates of comorbidities were observed in the PRISm compared to COPD: systemic arterial hypertension (46 versus 15%; *p* = 0.202), depression (23 versus 30%; *p* = 1.000), anxiety (23 versus 7%; *p* = 0.593), diabetes (38 versus 7%; *p* = 0.160) and history of post-COVID-19 symptoms (23 versus 7%; *p* = 0.593). Prescribed inhaled medications comparing PRISm and COPD patients included as-needed short-acting bronchodilators (69 versus 76%; *p* = 0.100), single long-acting bronchodilator (7 versus 46%; *p *= 0.073), long-acting bronchodilator plus inhaled corticosteroids (76 versus 23%; *p* = 0.006), and dual long-acting bronchodilators plus inhaled corticosteroids (0 vs 7%; *p* = 0.100).

**Table 1:** Clinical and physiological characteristics of the included participants

Variables	PRISm (*n* = 13)	COPD (*n* = 13)	Controls (*n* = 13)
Age, years	62.4 ± 7.6	58.8 ± 8.3	62.4 ± 5.1
Gender, female, n (%)	11 (84)	10 (76)	9 (69)
Height, m	1.57 ± 0.07	1.60 ± 0.08	1.61 ± 0.08
BMI, kg/m^2^	28.1 ± 7.5	25.5 ± 2.8	26.7 ± 3.7
Current smokers, n (%)	10 (77) ^***^	4 (31)	0
Smoking history, pack-years	70 [40–109]	36 [24–61]	0
Exacerbations previous 12 m	0.5 [0.0–1.0]	0.0 [0.0–0.5]	–
mMRC (0–4), units	3.0 [1.0–3.0]	2.0 [1.5–3.0]	–
mMRC ≥ 2, n (%)	9 (69)	10 (76)	–
CAT (0–40), units	24.1 ± 7.9	22.2 ± 12.0	–
CAT ≥ 10, n(%)	11 (84)	9 (69)	–
Only FEV_1_ < 80% pred, n (%)	1 (7)	5 (38) ^**^	0 (0)
Only FVC < 80% pred, n (%)	0 (0)	0 (0)	0 (0)
FEV_1_&FVC < 80% pred, n (%)	12 (92)^*^^,^^***^	3 (23)	0 (0)
COPD stages by GOLD			
1, n (%)	–	5 (38)	–
2, n (%)	–	8 (62)	–
Pulmonary Function			
FEV_1_, L (% pred)	1.72 ± 0.27^*^ (71 ± 6)^*^	1.76 ± 0.42^**^ (72 ± 13) ^**^	2.47 ± 0.52 (97 ± 11)
FEV_1,_ z score	−1.89 ± 0.41^*^	−1.87 ± 0.90^**^	−0.18 ± 0.76
FVC, L (% pred)	2.26 ± 0.42^*,^^***^ (73 ± 6)^*^^,^^***^	2.91 ± 0.61 (94 ± 14)	3.02 ± 0.64 (96 ± 11)
FVC, z score	−1.80 ± 0.48^*,^^***^	−0.38 ± 0.95	−0.30 ± 0.75
FEV_1_/FVC, (% pred)	0.76 ± 0.05^***^ (96 ± 5) ^***^	0.60 ± 0.04^**^ (75 ± 5) ^**^	0.79 ± 0.02 (101 ± 3)
FEV_1_/FVC, z score	−0.32 ± 0.69^***^	−2.49 ± 0.52^**^	0.12 ± 0.38
${\mathrm{FEF}}_{25-75\mathrm{\%}}$, L/s (% pred)	1.42 ± 0.36^***^ (65 ± 14) ^***^	0.79 ± 0.27 (34 ± 9)	–
${\mathrm{FEF}}_{25-75\mathrm{\%}}$, z score	−1.09 ± 0.48	−2.45 ± 0.59	–
TLC, L (% pred)	4.90 ± 0.92^***^ (96 ± 8) ^***^	5.93 ± 1.10 (119 ± 17)	–
TLC, z score	−0.28 ± 0.70^***^	1.48 ± 1.36	–
TLC > ULN, n (%)	0 (0) ^***^	6 (46)	–
VC, L (% pred)	2.28 ± 0.44 (68 ± 6) ^***^	2.96 ± 0.59 (88 ± 12)	–
VC, z score	−2.39 ± 0.51^***^	−0.92 ± 1.00	–
VC-FVC, L	0.00 [0.00–0.03]	0.00 [0.00–0.07]	–
FRC, L (% pred)	2.87 [2.43–3.84] (114 ± 24) ^***^	3.48 [3.04–4.96] (152 ± 37)	–
FRC, z score	0.64 ± 1.07^***^	2.11 ± 1.34	–
RV, L (% pred)	2.35 ± 0.70 (134 ± 27) ^***^	3.00 ± 1.00 (188 ± 62)	–
RV, z score	1.09 ± 0.90^***^	2.52 ± 1.64	–
RV > ULN, n (%)	3 (23) ^***^	10 (77)	–
RV/TLC, (% pred)	0.47 ± 0.09 (140 ± 25)	0.50 ± 0.09 (156 ± 34)	–
RV/TLC, z score	1.87 ± 1.21	2.53 ± 1.46	–
RV/TLC > ULN, n (%)	9 (69)	9 (69)	–
IC, L (% pred)	1.80 ± 0.40 (75 ± 13)	2.00 ± 0.40 (84 ± 23)	–
IC, z score	−1.25 ± 0.68	−0.81 ± 1.21	–
Raw, kPa/Ls^−1^ (% pred)	0.52 ± 0.21 (235 [189–272])	0.55 ± 0.33 (221 [150–287])	–
DL_CO_, mmol/min/kPa (% pred)	4.78 ± 1.24^***^ (73 ± 17) ^***^	3.60 ± 0.96 (55 ± 17)	–
DL_CO_, z score	−1.97 ± 1.25^***^	−3.65 ± 1.76	–
K_CO_, mmol/min/kPa/L (% pred)	1.13 [0.98–1.45] ^***^ (81 [67–102])^***^	0.78 [0.63–1.07] (52 [42–72])	–
K_CO_, z score	−1.31 [−2.16; 0.15]^***^	−3.63 [−4.49; –1.98]	−
VA, L (% pred)	4.04 ± 0.84 (87 ± 12)^***^	4.60 ± 0.72 (100 ± 15)	–
VA, z score	−1.11 ± 1.15^***^	0.04 ± 1.21	–
VA/TLC	0.82 ± 0.10	0.79 ± 0.12	–
VA/TLC < 0.80, n (%)	6 (46)	5 (38)	–

BMI body mass index, mMRC Modified Medical Research Council dyspnea scale, CAT COPD assessment test, FEV_1_ forced expired volume in first second, FVC forced vital capacity, ULN upper limit of normal, GOLD Global Initiative for Obstructive Lung Disease, % pred percentage of predicted, FEF_25–75%_ forced expiratory flow between 25 and 75% of forced vital capacity, TLC total lung capacity, VC vital capacity, FRC functional residual capacity, RV residual volume, IC inspiratory capacity, Raw airway resistance, DL_CO_ lung diffusing capacity for carbon monoxide, K_CO_ carbon monoxide transfer coefficient, VA alveolar volume

Data are presented as mean ± SD or median [25^th^-75^th^ centile], unless otherwise stated

^*^P < 0.05 for PRISm patients *versus* controls

^**^P < 0.05 for COPD patients *versus* controls

^***^P < 0.05 for PRISm *versus* COPD patients

PRISm and COPD groups had equivalently reduced FEV_1_ compared to controls. While FVC was lower in the PRISm group compared to the other two groups, maximal mid-expiratory flows were most reduced in the COPD group. Plethysmography and DL_CO_ measurements were only available for patients. Airway resistance was increased in both groups. On average, static lung volumes (functional residual capacity (FRC), residual volume (RV), RV/TLC) were above the upper limit of normal (ULN) in the COPD group, which were significantly higher compared to the PRISm group. This later group, in turn, also presented with the average RV/TLC ratio above the ULN, explaining the PRISm pattern in the context of a TLC within the normal range. Furthermore, a high proportion of smokers in both groups presented with RV/TLC ratio above the ULN (⁓69%) and alveolar volume (VA)/TLC ratio below 0.8 (⁓40%), both indexes indicative of small airway dysfunction (Neder et al. 2019, 2023). DL_CO_ and carbon monoxide coefficient of transfer (K_CO_) were reduced in both groups, and significantly lower in COPD compared to PRISm (Table 1).

The proportion of subjects meeting any objective criteria for a maximal exercise test was 9 (69%), 12 (92%), and 12 (92%) in the PRISm, COPD, and control groups, respectively (*p* > 0.05). The PRISm group exhibited the lowest peak V̇O_2_ and exercise tolerance (peak work rate) among the three groups. Peak ventilatory reserve (1- ⩒E/eMVV) and peak VT/IC ratio did not differ between PRISm and controls (Table 2), which were significantly different compared to patients with COPD. Same results were observed comparing only patients who achieved at least one criterion for maximal exercise (Table 3).

**Table 2:** Metabolic, ventilatory, cardiac, and sensory responses to cycling incremental cardiopulmonary exercise testing comparing patients with PRISm, COPD and control subjects

Variables	PRISm (*n* = 13)	COPD (*n* = 13)	Controls (*n* = 13)
WR, Watts (% pred)	51 ± 20^*^ (42 ± 16)^*^^,^^***^	70 ± 20^**^ (68 ± 11) ^**^	105 ± 27 (94 ± 17)
V̇O_2_, L/min (% pred)	0.93 ± 0.33^*^ (66 [58–86])^*^	1.14 ± 0.23^**^ (82 [74–96]) ^**^	1.56 ± 0.44 (108 [102–123])
V̇O_2_, mL/kg/min	12.3 ± 2.5^*^^,^^***^	18.2 ± 3.5^**^	22.1 ± 4.2
V̇O_2_/WR slope, mL/min/W	10.2 [6.7–11.8]	10.0 [9.2–11.0]	12.0 [9.7–13.4]
V̇CO_2_, L/min	0.93 ± 0.32^*^	1.30 ± 0.33^**^	1.74 ± 0.48
RER	1.00 ± 0.13^*^	1.09 ± 0.09	1.13 ± 0.08
V̇E, L/min	35.7 ± 10.2^*,^^***^	51.7 ± 17.5	56.7 ± 17.6
Ventilatory Reserve	0.50 ± 0.15^***^	0.28 ± 0.15^**^	0.43 ± 0.12
ƒ, breaths/min	30 ± 4	34 ± 4	35 ± 8
VT, L/min	1.07 ± 0.27^*^	1.46 ± 0.54	1.70 ± 0.50
ƒ/VT, %	27 [23–37]	23 [16–36]	20 [16–25]
V̇E/V̇CO_2_	36.0 ± 5.4	38.0 ± 4.8^**^	32.1 ± 3.4
V̇E/V̇CO_2_ slope	31.7 ± 4.6	33.3 ± 5.4	29.8 ± 3.3
V̇E/V̇CO_2_ nadir	35.6 ± 5.3^*^	36.7 ± 5.4^**^	29.0 ± 3.9
IC, L/min	1.83 [1.68–2.13]^*^	1.82 [1.39–2.58] ^**^	2.38 [2.12–2.75]
VT/IC, L/min	0.58 ± 0.12^***^	0.74 ± 0.13^**^	0.64 ± 0.09
HR, bpm (% pred)	115 ± 25^*^ (75 ± 16)^*^	132 ± 18 (82 ± 9)	145 ± 13 (93 ± 9)
O_2_ pulse, mL/beats	7.6 [5.8–9.8]	10.0 [7.7–10.7]	10.1 [8.8–11.8]
SBP, mmHg	140 ± 27	152 ± 15	–
DBP, mmHg	86 ± 12	91 ± 7	–
SpO_2_, %	95 ± 2	95 ± 1	97 ± 1
Borg dyspnoea , units	4.0 [2.0–6.0]	4.5 [3.0–6.7]	5.0 [3.0–8.0]
Borg leg discomfort, units	4.0 [3.0–7.0]	6.0 [3.2–7.7]	7.0 [3.0–9.5]

All parameters refer to peak exercise values unless otherwise stated

*WR* work rate %, *pred* percentage of predicted, *V̇O*_2_ oxygen uptake, *V̇CO*_2_ carbon dioxide output, *RER* respiratory exchange ratio, *V̇E* ventilation, ƒ breathing frequency, *VT* tidal volume, *IC* inspiratory capacity, Δ change from rest; *HR* heart rate, *O*_*2*_ oxygen, *SBP*, systolic blood pressure, *DBP* diastolic blood pressure, *SpO*_*2*_ oxyhemoglobin saturation by pulse oximetry

Data are presented as mean ± SD or median [25^th^-75^th^ centile], unless otherwise stated

^*^P < 0.05 for PRISm patients *versus* controls

^**^P < 0.05 for COPD patients *versus* controls

^***^P < 0.05 for PRISm *versus* COPD patients

**Table 3:** Metabolic, ventilatory, cardiac, and sensory responses to cycling incremental cardiopulmonary exercise testing of participants who met the criteria for maximal exercise according to international guidelines

Variables	PRISm (*n* = 9)	COPD (*n* = 12)	Controls (*n* = 12)
WR, Watts (% pred)	55 ± 17^*^ (48 [33–55])^*^	71 ± 20^**^ (72 [63–76]) ^**^	108 ± 26 (94 [82–101])
V̇O_2_, L/min (% pred)	0.95 ± 0.25^*^ (74 [62–86])^*^	1.14 ± 0.24^**^ (82 [72–98]) ^**^	1.58 ± 0.44 (109 [101–127])
V̇O_2_, mL/kg/min	12.2 ± 1.7^*,^^***^	18.5 ± 3.5^**^	22.5 ± 4.2
V̇O_2_/WR slope, mL/min/W	9.7 ± 2.1	10.5 ± 2.1	11.4 ± 2.2
V̇CO_2_, L/min	0.99 ± 0.25^*^	1.31 ± 0.34^**^	1.79 ± 0.47
RER	1.04 ± 0.14	1.10 ± 0.07	1.14 ± 0.06
V̇E, L/min	39.5 [34.0–42.2]^*^	46.6 [42.2–69.3]	58.8 [41.7–66.7]
Ventilatory Reserve	0.48 ± 0.14^***^	0.26 ± 0.14^**^	0.43 ± 0.12
ƒ, breaths/min	31 ± 3	34 ± 5	34 ± 8
VT, L/min	1.14 [0.89–1.24]^*^	1.34 [1.08–2.12]	1.60 [1.43–1.95]
ƒ/VT, %	30 ± 8	25 ± 11	20 ± 7
V̇E/V̇CO_2_	35.3 ± 6.1	38.4 ± 4.7^**^	32.2 ± 3.5
V̇E/V̇CO_2_ slope	31.6 ± 4.7	34.0 ± 5.2	30.1 ± 3.3
V̇E/V̇CO_2_ nadir	35.0 ± 5.8^*^	37.1 ± 5.4^**^	28.9 ± 4.0
IC, L/min	1.83 [1.68–1.99]^*^	1.94 [1.37–2.69]^**^	2.42 [2.17–2.87]
VT/IC, L/min	0.60 ± 0.07^***^	0.76 ± 0.12^**^	0.65 ± 0.08
HR, bpm (% pred)	119 ± 21^*^ (78 ± 14)^*^	134 ± 17 (82 ± 9)^**^	147 ± 12 (94 ± 8)
O_2_ pulse, mL/beats	7.9 ± 1.5	9.2 ± 1.7	10.8 ± 3.5
SBP, mmHg	144 ± 32	151 ± 16	–
DBP, mmHg	87 ± 14	90 ± 7	–
SpO_2_, %	95 ± 2	95 ± 1	97 ± 1
Borg dyspnoea , units	3.0 [0.5–4.5]	4.0 [3.0–6.0]	5.0 [3.0–8.5]
Borg leg discomfort, units	4.0 [2.5–6.0]	5.0 [3.0–7.0]	7.0 [4.7–9.7]

All parameters refer to peak exercise values unless otherwise stated

Data are presented as mean ± SD or median [25^th^-75^th^ centile], unless otherwise stated

*WR* work rate %, *pred* percentage of predicted, *V̇O*_2_ oxygen uptake, *V̇CO*_2_ carbon dioxide output, *RER* respiratory exchange ratio, *V̇E* ventilation, *ƒ* breathing frequency, *VT* tidal volume, *IC* inspiratory capacity, Δ change from rest, *HR* heart rate, *O*_*2*_ oxygen, *SBP* systolic blood pressure, *DBP* diastolic blood pressure, *SpO*_*2*_ oxyhemoglobin saturation by pulse oximetry

^*^P < 0.05 for PRISm patients *versus* controls

^**^P < 0.05 for COPD patients *versus* controls

^***^P < 0.05 for PRISm *versus* COPD patients

**Fig. 2:**
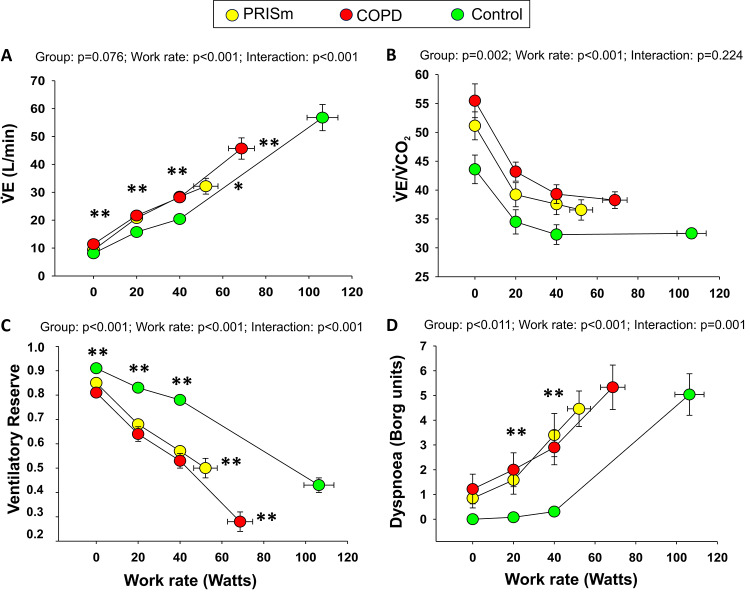
Ventilatory responses and dyspnoea perception during incremental exercise contrasting patients with preserved ratio impaired spirometry (PRISm) (*N* = 13), COPD (*N* = 13) and healthy controls (*N* = 13). Data are presented as mean ± standard error. *VE* ventilation, *VCO*_*2*_ carbon dioxide output. ^*^p < 0.05 for PRISm patients *versus* controls at a given work rate ^**^p < 0.05 for COPD patients *versus* controls at a given work rate ^***^p < 0.05 for PRISm *versus* COPD patients at a given work rate. When the symbols are lateral to the circles, they refer to comparisons at peak work rate (which differ across groups)

**Fig. 3:**
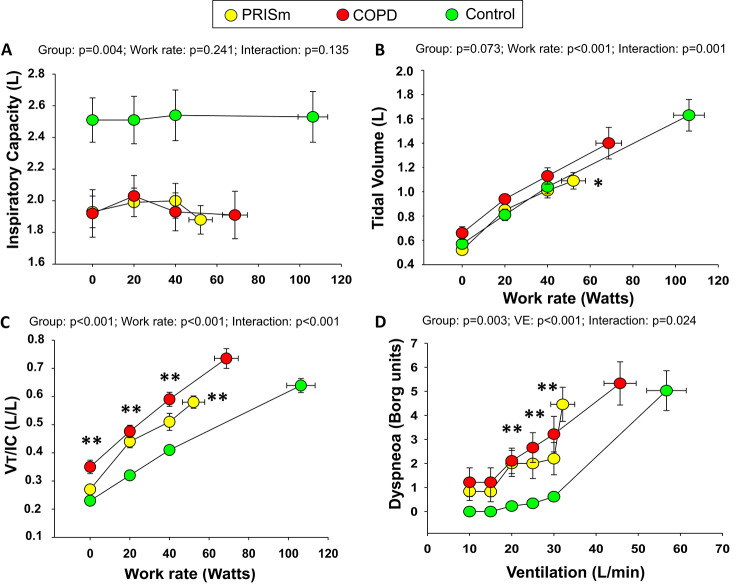
Ventilatory mechanics and dyspnoea perception during incremental exercise, in patients with PRISm (*N* = 13), COPD (*N* = 13) and healthy controls (*N* = 13). Data are presented as mean ± standard error. *VT* tidal volume, *IC* inspiratory capacity. ^*^p < 0.05 for PRISm patients *versus* controls at a given work rate (or ventilation) ^**^p < 0.05 for COPD patients *versus* controls at a given work rate (or ventilation). ^***^p < 0.05 for PRISm *versus* COPD patients at a given work rate (or ventilation). When the symbols are lateral to the circles, they refer to comparisons at peak work rate (which differ across groups)

**Fig. 4:**
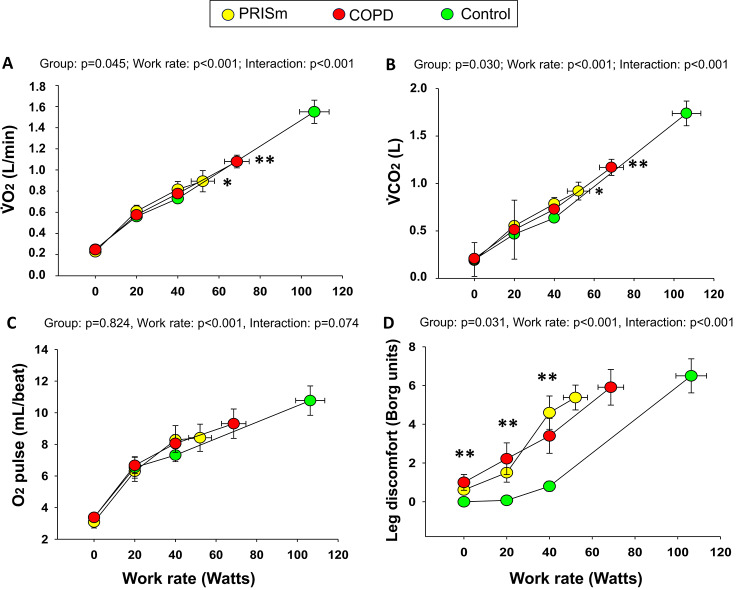
Metabolic and leg discomfort perception during incremental exercise contrasting patients with PRISm (*N* = 13), COPD (*N* = 13) and healthy control participants (*N* = 13). Data are presented as mean ± standard error. *O*_*2*_ oxygen, *V̇O*_*2*_ oxygen uptake, *V̇CO*_*2*_, carbon dioxide output; * *p* < 0.05 for PRISm patients *versus* controls at a given work rate. **p < 0.05 for COPD patients *versus* controls at a given work rate. ***p < 0.05 for PRISm *versus* COPD patients at a given work rate. When the symbols are lateral to the circles, they refer to comparisons at peak work rate (which differ across groups)

Patients presented with significantly higher V̇E for a given workload compared to controls (Fig. 2, Panel A), although the kinetics of V̇E/V̇CO_2_ difference was non-significant (p = 0.224) for the interaction between groups and work rate in the generalized estimating equation model (Fig. 2, Panel B). This higher exertional ventilatory demand was confirmed by increased V̇E/V̇CO_2_ nadir in the smoker groups (Table 2). It follows that higher ventilation for reduced FEV_1_ resulted in significantly lower ventilatory reserves in patients (Fig. 2, Panel C) and higher dyspnoea perception for a given submaximal workload compared to controls (Fig. 2, Panel D). A trend towards lower IC (Fig. 3, Panel A) for a similar VT (Fig. 3, Panel B) resulted in significantly higher VT/IC ratios (Fig. 3, Panel C) during submaximal exercise in patients. Accordingly, a higher dyspnoea perception at isoventilation (Fig. 3, Panel D) was observed in these groups compared to controls. Finally, the slopes of ⩒O_2_ (Fig. 4, Panel A), ⩒CO_2_ (Fig. 4, Panel B) and oxygen pulse (Fig. 4, Panel C) versus work rate were not different, indicating a similar metabolic demand during the exercise in the three groups. Therefore, the peak V̇O_2_ was lower in patients because they stopped the progressive exercise earlier. Leg discomfort, in turn, was greater for a given workload in patients compared to controls (Fig. 4, Panel D).

## Discussion

The main findings of the present study were: (1) the combination of lower resting FEV_1_ and higher exertional ventilation resulted in lower ventilatory reserve (Berton et al. 2023) and higher VT/IC ratios (Hijleh et al. 2024) during submaximal exercise in patients compared to controls; (2) accordingly, higher dyspnoea perception was observed for a given submaximal work rate and for a given level of ventilation in both groups of patients (Berton et al. 2021; Neder et al. 2020); (3) culminating in early exercise cessation at lower workloads compared to healthy controls. Of note, smokers with PRISm showed similar responses to exercise compared to COPD patients with equivalent reduced FEV_1_, i.e., GOLD stages 1 and 2. Although the current study was not designed to detect differences between PRISm and COPD, we believe that any eventual statistically significant differences, based on the trends observed in our data, would likely lack clinical relevance. In fact, in a previous study involving a comparable sample size of mild COPD patients, a comparable magnitude of differences in V̇E, V̇E/V̇CO_2_, and VT was observed before and after a specific intervention, which was ultimately deemed ineffective in producing meaningful improvements in exercise responses (Hirai et al. 2017).

The seminal work of Faisal et al in 2016 exposed the common mechanisms of dyspnoea and exercise intolerance in patients with divergent baseline chronic respiratory diseases like COPD and fibrosing interstitial lung disease (ILD) (Faisal et al. 2016). Since then, increasing evidence has shown that the main factor contributing to exercise intolerance in individuals with chronic cardiopulmonary disease is a mismatch between the demand for ventilation and the capacity of the thoracic-pulmonary system to meet that demand. This imbalance leads to a cascade of physiological events that culminate in higher breathlessness and the inability to sustain exercise (James et al. 2025). Here again and in agreement with previous work (Phillips et al. 2024), we showed that regardless of the primary mechanism of impaired resting lung function (a nonspecific ventilatory defect showing reduced FEV_1_ with preserved FEV_1_/FVC ratio and TLC in PRISm versus a “classical” obstruction with a reduced FEV_1_/FVC ratio in COPD), an excessive ventilation for a given workload (Figure 2A) resulted in lower ventilatory reserve (Figure 2C), which translated into higher dyspnoea perception during submaximal exercise compared to controls (Figure 2D). This mismatch between ventilatory demand and capacity resulting in higher dyspnoea burden has been previously described in patients with COPD (Berton et al. 2023) and ILD (Plachi et al. 2024), including mild ILD (Smyth et al. 2023). Similarly, high V̇E/V̇CO_2_ nadir was consistently associated with increased dyspnoea and reduced peak V̇O_2_, regardless of the severity of COPD (Phillips et al. 2022). It is not possible to determine whether the increased ventilatory demand is due to ventilatory inefficiency (indicated by an increased dead space/VT ratio) and/or alveolar hyperventilation (characterized by low arterial carbon dioxide pressure), or if both factors contributed equally, because arterial gas pressure measurements were not conducted (Neder et al. 2024). In line with mild COPD (James et al. 2021; Phillips et al. 2021) and ILD (Smyth et al. 2023), we can infer that impaired gas-exchange capacity (exposed by a low DL_CO_) may have contributed to some extent. The high percentage of smokers with abnormal RV/TLC and VA/TLC ratios also indicates some level of small airway dysfunction and ventilation inhomogeneity in both groups of smokers (Davis et al. 2016) (Table 1). The ventilation-perfusion mismatch requires a greater total minute ventilation to achieve the same level of carbon dioxide removal as a healthy individual (Petersson & Glenny 2014), and therefore increases the work of breathing.

Excessive ventilation during exercise may accelerate the onset of inspiratory mechanical constraints in patients with only mild respiratory impairment (James et al. 2025). Accordingly, higher ratios of VT/IC corresponded to greater perceptions of dyspnoea at a given level of ventilation in both groups of smokers. An increased dyspnoea -V̇E slope is an indicator of abnormal ventilatory mechanics, despite a preserved ventilatory reserve at peak exercise (greater than 15%) or before reaching the traditional critical VT/IC threshold (>0.7) for further VT expansion (Berton et al. 2021; Neder et al. 2020). Notably, ventilatory differences in the PRISm versus the control group were only observed during submaximal exercise and would be overlooked if only peak values were compared. This reinforces that a single-point assessment of any physiological response is inherently limited in exposing the underpinnings of dyspnoea that develops gradually and worsens in response to submaximal phenomena, i.e., high submaximal ventilatory demands coupled with mechanical abnormalities may elicit limiting dyspnoea well before the attainment of a critical peak value (James et al. 2025).

Another finding of the present study was the lower values of key peak exercise parameters observed in patients with PRISm compared to those with COPD. The first group presented with lower peak aerobic capacity, peak work rate, VE/eMVV and VT/IC (Table 2). Even assessing only subjects who achieved any criteria indicative of maximal exercise, most of these differences continue to be observed (Table 3). We speculate that patients with PRISm may exhibit lower motivation, leading to suboptimal effort, as well as decreased levels of daily physical activity and/or peripheral muscle function. These factors could potentially impact their exercise performance. Unfortunately, detailed data on physical activity levels and peripheral muscle function were not systematically collected. The lower exercise capacity observed in PRISm patients compared with COPD remains to be confirmed in future studies, including holistic assessments of psychological factors, daily physical activity, and peripheral muscle function. Notwithstanding, both groups of smokers reported greater sensations of lower limb discomfort compared to the controls (Figure 4, Panel D), indicating the same degree of lower limb muscle dysfunction (Maltais et al. 2014).

Finally, it is worth mentioning that the PRISm pattern observed in our participants appears to be caused by a combination of air trapping in the context of preserved TLC. All individuals with PRISm had TLC within normal ranges whereas almost half (6/13) of the COPD subjects presented with thoracic hyperinflation (TLC> upper limit of normal). The reduced mean values of DL_CO_ and K_CO_ also indicate some degree of alveolar dysfunction in these patients. The increased RV/TLC was also found in the PRISm group by Phillips and colleagues (Phillips et al. 2024), where, however, 15% of the PRISm sample presented with restriction (reduced TLC). Phillips *et al* also demonstrated that the extension of emphysema (low-attenuation areas of the lung below -950 Hounsfield units) or the probability of functional small airway disease derived by chest computed tomography was similar in PRISm versus controls, and both groups were better compared to COPD. While an increase in RV or RV/TLC above the 95^th^ percentile may indicate air trapping due to the presence of airway obstruction, this finding may also reflect muscle weakness, suboptimal effort and restrictive processes when TLC is proportionally more reduced than RV (Stanojevic et al. 2022). In addition, the prescription patterns for inhaled treatments differed between patients with PRISm and those with COPD. A higher percentage of PRISm patients received corticosteroids, while fewer were prescribed long-acting bronchodilators. The reason for this difference is unclear. Although patients with a prior or suspected history of asthma or other forms of transient airway hyperresponsiveness were systematically excluded from the study, we cannot draw conclusions about the effects of treatment modifications or changes in resting functional patterns over time, as our analysis was cross-sectional. Altogether, these findings expose that the PRISm pattern is more complex and heterogeneous than initially thought (Knox-Brown et al. 2022).

We must recognize that this is a small-sample study with participants recruited from a single center, with a notable female predominance. Paradoxically, the former characteristic also represent one of its major strength. Different from extensive population-based studies and/or large cohorts of smokers, we had the opportunity to individually review each included and excluded participant with PRISm. Therefore, this study offered a clinical rather than an epidemiological perspective. Although restricted to a single study visit, all participants performed a detailed CPET with state-of-the-art methodology to assess the current understanding of the physiological bases of dyspnoea  (Berton et al. 2021; James et al. 2025; Stickland et al. 2022). Unfortunately, controls did not perform lung volume measurements by plethysmography, precluding the contrast of other useful indexes of ventilatory mechanics during exercise (end-inspiratory and expiratory lung volumes/TLC) with those of the patients.

## Conclusion

Smokers with PRISm exhibited significantly greater dyspnoea and reduced exercise capacity compared to healthy controls. These adverse clinical outcomes appear to be driven by a mismatch between a higher ventilatory demand and a lower ventilatory capacity during submaximal exertion.

## Data Availability

The data that support the findings of this study are available from the corresponding author upon reasonable request.

## Abbreviations

BMI: Body mass index
CAT: COPD assessment test
COPD: Chronic Obstructive Pulmonary Disease
CPET: Cardiopulmonary exercise test
DBP: Diastolic blood pressure
DL_CO_: Lung diffusing capacity for carbon monoxide
eMVV: Estimated maximal voluntary ventilation
ƒ: Breathing frequency
FEF_25–75%_: Forced expiratory flow between 25 and 75% of forced vital capacity
FEV_1_: Forced expired volume in first second
FRC: Functional residual capacity
FVC: Forced vital capacity
GOLD: Global Initiative for Chronic Obstructive Lung Disease
HR: Heart rate
IC: Inspiratory capacity
K_CO_: Carbon monoxide transfer coefficient
LLN: Lower limit of normal
mMRC: Modified Medical Research Council dyspnoea scale
O_2_: Oxygen
PRISm: Preserved Ratio Impaired Spirometry
RER: Respiratory exchange ratio
RV: Residual volume
SBP: Systolic blood pressure
SpO_2_: Oxyhemoglobin saturation by pulse oximetry
TLC: Total lung capacity
ULN: Upper limit of normal
VA: Alveolar volume
VC: Vital capacity
V̇CO_2_: Carbon dioxide output
V̇E: Ventilation
V̇O_2_: Oxygen uptake
VT: Tidal volume
WR: Work rate
